# Validation of the Swedish Multiple Sclerosis registry for pediatric-onset multiple sclerosis

**DOI:** 10.1177/20552173251314118

**Published:** 2025-02-09

**Authors:** Fredrik Sandesjö, Peter Alping, Katharina Fink, Ronny Wickström, Fredrik Piehl, Thomas Frisell, Kyla A McKay

**Affiliations:** Neuropediatric Unit, 167725 Astrid Lindgren Children's Hospital, Karolinska University Hospital, Stockholm, Sweden; Department of Women's and Children's Health, 27106 Karolinska Institutet, Stockholm, Sweden; Division of Clinical Epidemiology, Department of Medicine, Solna, 27106 Karolinska Institutet, Stockholm, Sweden; Department of Clinical Neuroscience, 27106 Karolinska Institutet, Stockholm, Sweden; Center for Molecular Medicine, 27106 Karolinska Institutet, Stockholm, Sweden; Neuropediatric Unit, 167725 Astrid Lindgren Children's Hospital, Karolinska University Hospital, Stockholm, Sweden; Department of Women's and Children's Health, 27106 Karolinska Institutet, Stockholm, Sweden; Department of Clinical Neuroscience, 27106 Karolinska Institutet, Stockholm, Sweden; Center for Molecular Medicine, 27106 Karolinska Institutet, Stockholm, Sweden; Division of Clinical Epidemiology, Department of Medicine, Solna, 27106 Karolinska Institutet, Stockholm, Sweden; Department of Clinical Neuroscience, 27106 Karolinska Institutet, Stockholm, Sweden; Center for Molecular Medicine, 27106 Karolinska Institutet, Stockholm, Sweden

**Keywords:** MS, register, PoMS, treatment, therapy, children, real world, evidence, observational

## Abstract

Few controlled trials of disease-modifying therapies (DMTs) have been conducted on the pediatric-onset multiple sclerosis (PoMS) population, leading to extensive off-label use of therapies approved only for adults. This highlights the need for real-world evidence to guide clinical practice. Clinical registries can offer high-quality data, but limitations such as missing and erroneous information must be considered. This validation study compared Swedish Multiple Sclerosis registry data from 122 PoMS patients to medical records. Generally (≥89%), data were confirmed. However, missing data exceeded 30% for rituximab infusions, magnetic resonance imaging, and relapses. Overall, the registry provides valid, real-world data on DMT use in PoMS.

## Introduction

Multiple sclerosis (MS) is a chronic, inflammatory disease affecting the central nervous system.^1^ Although most patients are diagnosed as adults, 2–5% experience disease onset during childhood, referred to as pediatric-onset MS (PoMS).^2^ Pediatric-onset MS patients tend to exhibit more inflammatory disease activity than patients with adult-onset MS, particularly early in the disease course.^3,4^ Due to the exclusion of children from pivotal randomized controlled trials, most disease-modifying therapies (DMTs) approved for MS have not been formally approved for use in children. Thus, PoMS patients are primarily treated off-label based on guidelines extrapolated from the adult MS population.^5^

Sweden offers a prime setting for conducting epidemiological studies, with well-established, high-quality nationwide registers like the Swedish MS registry. The MS registry captures over 80% of prevalent MS cases in the country and offers the opportunity to analyze trends over large patient numbers and long observation periods.^6^ In 2019, the MS registry was validated through a comprehensive chart review of more than 3000 patients, including some pediatric-onset patients.^7,8^ Because the MS registry is not as widely used among child neurologists as adult neurologists, PoMS patients are often not registered until they reach adulthood (18 years) and see an adult neurologist who records their data retrospectively. This raises the concern that the high validity found in the overall population^7^ may not be generalizable to PoMS.

Our study aimed to determine whether the MS registry is also valid for the study of PoMS, with a focus on DMT use. If so, the registry can be used for observational, real-world studies of treatment persistence, long-term effectiveness, and safety in this population. Studies on these topics are scarce, consisting mostly of case series or smaller cohort studies. Thus, validated real-world data would add great value to future studies of PoMS.

## Methodology

As part of the COMBAT-MS study,^7,8^ seven Swedish university clinics were instructed to review the medical charts for a selection of their patients enrolled in the MS registry. The inclusion criteria for each individual were: (1) Treated with a DMT at the university clinic; (2) Starting a first or second DMT between January 1, 2011, and December 31, 2016 (the inclusion therapy); and (3) Relapsing-remitting MS at the start of the inclusion therapy. Using medical charts as the ‘gold standard’, information collected in the registry was reviewed (and updated in the registry if missing/erroneous) for the following variables: onset date, relapses (date and description [monofocal optic neuritis, afferent non-optic neuritis, and/or monofocal]), magnetic resonance imaging (MRI) (date of scan, total number of T2 lesions and the total number of contrast-enhancing lesions in the brain and spinal cord), Expanded Disability Status Scale (EDSS) (date and score), therapy (product, start, and stop dates), and rituximab infusion dates (Table S1). The focus on rituximab infusions was motivated by the widespread use in Sweden, where the majority of MS patients are prescribed this DMT.^9^

For the present study, we used the same datasets but restricted to persons with an onset of MS before age 18 years. Following comparison to the medical chart, registry data were considered confirmed, having changed date or data, removed (if missing from medical charts), or missing (if in medical charts but not registry). Results were presented for the full cohort and stratified by treatment onset age (<18 years and ≥18 years). We tested for differences between the strata using Fisher's exact test (significance level α = 0.05).

The COMBAT-MS study is approved by the regional ethical review board in Stockholm (2017/32–31/4).

## Results

We identified 122 persons with PoMS in the COMBAT cohort; characteristics are summarized in Table 1. Age distributions at onset and the start of the first treatment are presented in Figure S1.

**Table 1.** Characteristics of the validation cohort consisting of 122 pediatric-onset multiple sclerosis patients.

	Overall (*N* = 122)
Age at onset	
Median [IQR]	16.1 [2.4]
Sex	
Female	88 (72.1%)
Male	34 (27.9%)
Age at first DMT start	
Median [IQR]	17.5 [3.0]
Age at first DMT start – dichotomized	
<18 years	73 (59.8%)
≥18 years	49 (40.2%)

IQR: interquartile range; DMT: disease-modifying therapy.

All persons with a PoMS onset in the registry were correctly identified as such. In addition, two more MS patients were identified as having a pediatric onset after chart review. Generally, a high proportion (≥89%) of the registry data was confirmed in the medical charts. The exception was MRI observations, where 18% of the data were changed based on medical chart review. Most (46/57, 81%) of these changes were related to missing data on the total number of T2 lesions. In only 11 MRI observations (4%), were existing data changed after chart review. The proportion of missing observations was low for onset date and therapy (≤6%), moderate for EDSS (10–15%), and higher for rituximab infusions, relapses, and MRI (29%–55%). Patients who started therapy before age 18 had comparable missing rates as those who started therapy after age 18 (Table 2).

**Table 2.** Number of observations in the MS registry before chart review and number and proportion of observations changed, removed, or missing after chart review.

Variable	Age at treatment start	Events recorded in register	Confirmed (%)	Changed date (%)	Changed data (%)	Removed (%)	Missing (%)
Onset date	Overall	120	111 (93)	0 (0)	9 (8)	0 (0)	2 (2)
	<18 years	72	66 (92)	0 (0)	6 (8)	0 (0)	1 (1)
	≥18 years	48	45 (94)	0 (0)	3 (6)	0 (0)	1 (2)
Therapy	Overall	255	233 (91)	4 (2)	9 (4)	9 (4)	15 (6)
	<18 years	155	143 (92)	2 (1)	4 (3)	6 (4)	9 (5)
	≥18 years	100	90 (90)	2 (2)	5 (5)	3 (3)	6 (6)
Rituximab infusions	Overall	103	100 (97)	0 (0)	1 (1)	2 (2)	113 (52)
	<18 years	58	56 (97)	0 (0)	1 (2)	1 (2)	57 (50)
	≥18 years	45	44 (98)	0 (0)	0 (0)	1 (2)	56 (55)
Relapse	Overall	108	99 (92)	2 (2)	6 (6)	1 (1)	58 (35)
	<18 years	63	59 (94)	2 (3)	2 (3)	0 (0)	40 (39)
	≥18 years	45	40 (89)	0 (0)	4 (9)	1 (2)	18 (29)
MRI	Overall	312	238 (76)	11 (4)	57 (18)	6 (2)	281 (47)
	<18 years	187	140 (75)	9 (5)	34 (18)	4 (2)	186 (50)
	≥18 years	125	98 (78)	2 (2)	23 (18)	2 (2)	95 (43)
EDSS	Overall	508	488 (96)	0 (0)	19 (4)	1 (0)	75 (13)
	<18 years	285	276 (97)	0 (0)	9 (3)	0 (0)	51 (15)
	≥18 years	223	212 (95)	0 (0)	10 (4)	1 (0)	24 (10)

EDSS: Expanded Disability Status Scale; MRI: magnetic resonance imaging; MS: multiple sclerosis.

Results are reported for the overall cohort and stratified by age at treatment start.

## Discussion

In this study, we aimed to determine whether the Swedish MS registry is valid for studying PoMS, with a focus on DMT use. Data on DMT use and EDSS had high validity, while the data quality was lower for relapses and MRI. Generally, all variables were recorded in the registry with high accuracy. However, there was missing data, particularly for rituximab infusions, relapses, and MRI scans. These findings are consistent with those observed in the broader MS population.^7^ The proportion of missing data was not different for those starting a DMT before or after age 18.

In conclusion, care should be taken when selecting variables for PoMS studies. For instance, using MS registry data to measure relapses may lead to underestimating events. However, in the context of therapy observations, ≥90% of the data were confirmed, and ≤6% were missing. These findings demonstrate that this variable is highly valid for the full PoMS cohort, regardless of age at treatment start, and valuable for studying treatment-related outcomes. Considering this, the Swedish MS registry can be used to provide valid, longitudinal, real-world information on a large PoMS population, which could, in turn, influence clinical practice.

## Supplemental Material

sj-docx-1-mso-10.1177_20552173251314118 - Supplemental material for Validation of the Swedish Multiple Sclerosis registry for pediatric-onset multiple sclerosis Supplemental material, sj-docx-1-mso-10.1177_20552173251314118 for Validation of the Swedish Multiple Sclerosis registry for pediatric-onset multiple sclerosis by Fredrik Sandesjö, Peter Alping, Katharina Fink, Ronny Wickström, Fredrik Piehl, Thomas Frisell and Kyla A McKay in Multiple Sclerosis Journal – Experimental, Translational and Clinical

sj-docx-2-mso-10.1177_20552173251314118 - Supplemental material for Validation of the Swedish Multiple Sclerosis registry for pediatric-onset multiple sclerosis Supplemental material, sj-docx-2-mso-10.1177_20552173251314118 for Validation of the Swedish Multiple Sclerosis registry for pediatric-onset multiple sclerosis by Fredrik Sandesjö, Peter Alping, Katharina Fink, Ronny Wickström, Fredrik Piehl, Thomas Frisell and Kyla A McKay in Multiple Sclerosis Journal – Experimental, Translational and Clinical

## Data Availability

Requests for sharing of de-identified data will be considered on reasonable request and in accordance with current legislation regarding protection of personal data. Code is available on request.
