# Supplementary material for: Validation of the Swedish Multiple Sclerosis registry for pediatric-onset multiple sclerosis
Source: Mult Scler J Exp Transl Clin. 2025 Feb 9;11(1):20552173251314118. doi: 10.1177/20552173251314118 (PMC11808745; doi:10.1177/20552173251314118)

**eFigure 1.** Age distribution of the validation cohort of 122 pediatric-onset multiple sclerosis patients at onset and first treatment start.


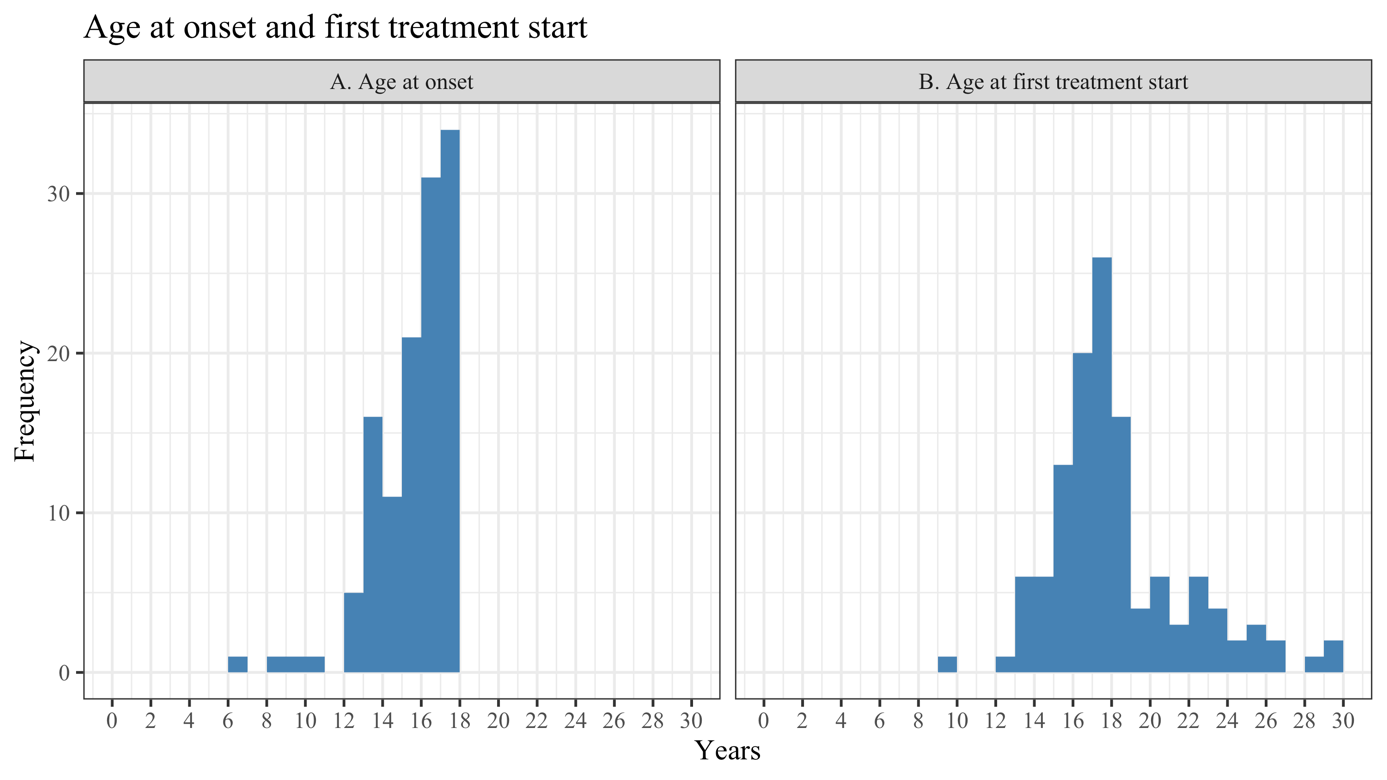

Supplement: sj-docx-1-mso-10.1177_20552173251314118 - Supplemental material for Validation of the Swedish Multiple Sclerosis registry for pediatric-onset multiple sclerosis [file sj-docx-1-mso-10.1177_20552173251314118.docx]
