# Supplementary material for: Validation of the Swedish Multiple Sclerosis registry for pediatric-onset multiple sclerosis
Source: Mult Scler J Exp Transl Clin. 2025 Feb 9;11(1):20552173251314118. doi: 10.1177/20552173251314118 (PMC11808745; doi:10.1177/20552173251314118)
Supplement: sj-docx-2-mso-10.1177_20552173251314118 - Supplemental material for Validation of the Swedish Multiple Sclerosis registry for pediatric-onset multiple sclerosis [file sj-docx-2-mso-10.1177_20552173251314118.docx]

**eTable 1.** Data and time periods reviewed

| Variable | Revied data | Time period |
| --- | --- | --- |
| Onset date | Onset date | From birth until 2016-12-31 |
| Therapy | Product, start and stop dates | From the first DMT start until 2016-12-31 |
| Rituximab infusion dates | Infusion dates | From the first rituximab infusion until 2016-12-31 |
| Relapses | Date and description (monofocal optic neuritis, afferent non-optic neuritis, and/or monofocal) | From 24 months before the start of inclusion therapy until 2016-12-31 |
| MRI | Date of scan, total number of T2 lesions and the total number of contrast-enhancing lesions in the brain and spinal cord | From the brain and spine scan closest in time before the start of inclusion therapy until 2016-12-31 |
| EDSS | Date and score | From 6 months before start of inclusion therapy until 2016-12-31 |
